# Preliminary Functional Screening of Autochthonous *Saccharomyces cerevisiae* from Mexican Cocoa Bean Fermentation for Traits Associated with Probiotic Potential

**DOI:** 10.3390/microorganisms14051153

**Published:** 2026-05-20

**Authors:** Aylin López-Palestino, Natali Hernández-Parada, Zorba Josué Hernández-Estrada, Oscar González-Ríos, Olaya Pirene Castellanos-Onorio, Rodrigo Alonso-Villegas, Aztrid Elena Estrada-Beltrán, Mirna Leonor Suárez-Quiroz, Claudia Yuritzi Figueroa-Hernández

**Affiliations:** 1 Tecnológico Nacional de México/Instituto Tecnológico de Veracruz, Unidad de Investigación y Desarrollo en Alimentos, M. A. de Quevedo 2779, Veracruz 91897, Mexico; d25024005@veracruz.tecnm.mx (A.L.-P.); d21020005@veracruz.tecnm.mx (N.H.-P.); zorba.he@veracruz.tecnm.mx (Z.J.H.-E.); oscar.gr@veracruz.tecnm.mx (O.G.-R.); olaya.co@veracruz.tecnm.mx (O.P.C.-O.); 2 Facultad de Ciencias Agrotecnológicas, Universidad Autónoma de Chihuahua, Av. Pascual Orozco s/n, Campus 1, Santo Niño, Chihuahua 31350, Mexico; ralonso@uach.mx; 3 Facultad de Enfermería y Nutriología, Universidad Autónoma de Chihuahua, Circuito Universitario s/n, Campus 2, Chihuahua 31125, Mexico; aeestrada@uach.mx

**Keywords:** cocoa bean fermentation, *Saccharomyces cerevisiae*, probiotic yeasts, gastrointestinal tolerance, hydrophobicity

## Abstract

Yeasts have attracted increasing attention as potential alternatives to traditional bacterial probiotic strains due to their physiological resilience and functional versatility. However, the probiotic potential of yeast strains associated with Mexican cocoa bean fermentation remains largely unexplored. Therefore, this study aimed to conduct a preliminary screening of physiological and surface-related traits associated with probiotic functionality in four autochthonous *Saccharomyces cerevisiae* strains (YCTA5, YCTA9, YCTA14, and YCTA16), previously isolated from cocoa fermentation, using *Saccharomyces boulardii* (Jarrow Formulas^®^) as a reference strain. Evaluated parameters included tolerance to temperature, pH, and bile salts; hemolytic activity; survival in vitro under gastrointestinal (GI) conditions; bile salt hydrolase activity; auto-aggregation; co-aggregation; hydrophobicity; and response to antifungal agents (fluconazole, ciclopirox, nystatin, and clotrimazole). All yeast strains grew at 37 °C and at pH 4–8 and showed no hemolytic activity. All strains exhibited high auto-aggregation (>70%) and hydrophobicity values ranging from 55 to 88%. In the coaggregation assay, strains YCTA9, YCTA14, and YCTA16 showed moderate interactions with *Escherichia coli*, *Bacillus cereus*, and *Listeria innocua*, with some combinations exceeding 50%. Nevertheless, none of the yeast strains exhibited measurable growth at pH 2; bile salt tolerance was limited to 0.1% Oxgall, and viability decreased by approximately 54–56% after simulated gastrointestinal transit. These findings indicate that although some strains exhibited promising surface-related properties, significant physiological constraints restrict their probiotic potential under the tested conditions. Therefore, the studied yeast strains should be regarded as preliminary candidates requiring further validation. This work provides a first-stage evaluation for identifying functional yeast strains from Mexican cocoa bean fermentation, serving as a basis for future in vitro and in vivo studies.

## 1. Introduction

In recent decades, the global food industry has undergone a paradigm shift primarily driven by increasing consumer awareness of the relationship between diet and health. This shift has resulted in a growing demand for functional foods—products that provide health benefits beyond basic nutrition—with particular emphasis on foods containing probiotic microorganisms [1]. Probiotics are defined as “live microorganisms which, when administered in adequate amounts, confer a health benefit to the host” [2]. Traditionally, the development of probiotic food products has focused on dairy matrices and bacterial groups, especially lactic acid bacteria (LAB) and *Bifidobacteria* [3]. Conversely, increasing interest in non-dairy alternatives has broadened the search for new microbial candidates and food matrices, including yeast and other microorganisms associated with fermented plant-based substrates [4,5,6,7,8].

Yeasts have increasingly attracted attention as microorganisms with probiotic potential, owing to their distinctive physiological characteristics. Unlike bacteria, yeasts are intrinsically resistant to antibiotics, which may allow their concomitant use during antibiotic therapy [9,10,11]. Their reported functional effects include pathogen interference, modulation of immune responses, and trophic interactions within the gut microbiota [9,12]. *Saccharomyces boulardii* is the best-established probiotic yeast, with documented clinical efficacy [9,13]. In contrast, increasing evidence suggests that other yeast species may also possess relevant functional traits. For example, strains of *Kluyveromyces marxianus* and *Pichia kudriavzevii* have shown tolerance to gastrointestinal-like stress conditions, high auto-aggregation and hydrophobicity, and bioactive properties, including cholesterol reduction, antioxidant, and immunomodulatory effects [14,15,16,17,18,19]. Likewise, some autochthonous *Saccharomyces cerevisiae* strains associated with fermented foods have shown tolerance to low pH, osmotic stress, and competitive microbial environments, supporting their evaluation as candidate strains for further gastrointestinal and functional studies [13,20].

Cocoa bean fermentation is a complex microbial ecosystem and a promising source of microorganisms with potential biotechnological applications [21,22,23,24]. This spontaneous process involves a dynamic succession of yeasts, lactic acid bacteria (LAB), and acetic acid bacteria (AAB), which collectively contribute to pulp degradation, ethanol production, acidification, heat generation, and the formation of volatile compounds associated with cocoa flavor development [25,26,27]. During fermentation, yeasts are exposed to progressively stressful conditions, including temperatures that may approach 50 °C, increased ethanol concentrations, and the accumulation of organic acids such as lactic, acetic, and citric acids [28,29]. These environmental pressures may favor strains with enhanced stress tolerance.

Although the technological role of cocoa-associated yeast in aroma and flavor formation is well established, its traits associated with probiotic functionality remain insufficiently explored. Recent studies have reported that some cocoa-derived yeasts may exhibit tolerance to gastrointestinal-like stress, auto-aggregation capacity, antioxidant activity, and other properties of potential functional relevance. Nonetheless, probiotic functionality cannot be inferred solely from ecological adaptation or technological performance, and targeted in vitro and in vivo screening is still required to identify promising candidates. This knowledge gap is particularly relevant in Mexican cocoa bean fermentations, where artisanal processing and environmental conditions may harbor unique and underexplored microbial diversity [21,22,23].

Therefore, this study aimed to perform an initial assessment of physiological and surface-related traits associated with probiotic functionality in four autochthonous *Saccharomyces cerevisiae* strains (YCTA5, YCTA9, YCTA14, and YCTA16) isolated from Mexican cocoa bean fermentation. Rather than claiming probiotic status, this work provides an exploratory evaluation to identify candidate yeast strains from a scarcely explored microbial niche. This approach is consistent with current strategies used to evaluate functional microorganisms before advanced in vitro, in vivo, and clinical validation.

## 2. Materials and Methods

### 2.1. Strain and Culture Conditions

The strains used in this study were obtained from the microorganism collection of the Grupo de Tecnología de Alimentos at the Unidad de Investigación y Desarrollo de Alimentos (UNIDA), Instituto Tecnológico de Veracruz, Veracruz, Mexico. Four strains were selected from a larger collection previously isolated from cocoa bean fermentation and subsequently characterized based on their taxonomic identity, availability, and relevance within the collection reported by Hernández-Parada [30]. Molecular identification was later confirmed by Hernández-Parada et al. [31] using amplification of the internal transcribed spacer (ITS) regions ITS1-5.8S-ITS2 with ITS4/ITS5 primers following Macrogen’s standard protocol (Seoul, Republic of Korea). Based on sequence analysis, the strains were identified as *Saccharomyces cerevisiae* (YCTA5, YCTA9, YCTA14, and YCTA16).

The bacterial strains used in the coaggregation assay were obtained from the Colección de Cultivos Microbianos (CDBB) at Centro de Investigación de Estudios Avanzados (CINVESTAV-IPN), Ciudad de México, Mexico. The indicator strains were *Escherichia coli* CDBB-B-964, *Listeria innocua* CDBB-B-1866, *Bacillus cereus* CDBB-B-1425, and *Micrococcus luteus* CDBB-B-72. The commercial probiotic yeast used for comparative purposes was *Saccharomyces boulardii* (Jarrow Formulas^®^, Los Angeles, CA, USA). Yeast strains were grown in YPD broth (MCD LAB, Tultitlán, Mexico) at 30 °C for 24 h, whereas bacterial indicator strains were grown in TSB medium (MCD LAB, Mexico) at 35 °C for 24 h.

### 2.2. Hemolytic Activity of Cocoa-Derived Yeast Strains

Hemolytic activity was evaluated as an exploratory safety-related trait following the method described by Menezes et al. (2020) [23]. Yeast strains were streaked onto plates containing 5% defibrinated sheep blood agar (MCD LAB, Mexico) and incubated at 30 °C for 48 h. Hemolytic activity was classified according to colony appearance and the surrounding halo as α-hemolysis (greenish zones), β-hemolysis (clear halo), or γ-hemolysis (no visible changes).

### 2.3. Tolerance to Temperature of Cocoa-Derived Yeast Strains

The growth response of yeast strains to different incubation temperatures was evaluated using a modified method based on Azhar and Munaim (2019) [32]. Yeast strains were cultivated in YPD broth with an initial inoculum of 8 × 10^6^ cells/mL, determined using a Neubauer counting chamber. Cultures were incubated at 20, 30, 37, 40, and 45 °C for 24 h. Growth under each condition was estimated by measuring the optical density at 600 nm (OD_600_) using a spectrophotometer, with uninoculated medium as the blank. OD_600_ values were used as a comparative indicator of biomass development during this assessment.

### 2.4. Tolerance to pH of Cocoa-Derived Yeast Strains

The growth responses of yeast strains to different conditions were evaluated using a modified method based on Azhar and Munaim (2019) [32]. Yeast cells were inoculated into YPD broth adjusted to pH 2, 4, 6, and 8 using 5 M HCl or 5 M NaOH as appropriate. Initial suspensions of 8 × 10^6^ cells/mL were prepared based on cell counts obtained with a Neubauer chamber. Cultures were incubated at 30 °C for 24 h. Growth was estimated by measuring OD_600_ with a spectrophotometer, using uninoculated broth as the blank. OD_600_ values served as a comparative indicator of biomass development in this assessment.

### 2.5. In Vitro Survival Under Simulated Gastrointestinal Conditions of Cocoa-Derived Yeast Strains

Survival of the yeast strains under simulated gastrointestinal tract (GIT) conditions was evaluated using an in vitro digestion model based on the INFOGEST 2.0 protocol described by Brodkorb et al. [33], with minor modifications. A three-stage model (oral, gastric, and intestinal) was performed using simulated salivary fluid (SSF), simulated gastric fluid (SGF), and simulated intestinal fluid (SIF). Stock electrolyte solutions for the simulated fluids were prepared according to the protocol using KCl (37.3 g/L), KH_2_PO_4_ (68 g/L), NaHCO_3_ (84 g/L), NaCl (117 g/L), MgCl_2_·6H_2_O (30.5 g/L), and (NH_4_)_2_CO_3_ (48 g/L). The pH of each phase was adjusted with 5 M HCl or 5 M NaOH as needed. Overnight yeast cultures were centrifuged (4000 rpm, 15 min, 4 °C), washed twice, and resuspended in phosphate buffer (pH 7). Cell suspensions were adjusted to OD_600_ = 0.9 ± 0.02, corresponding to approximately 1–2 × 10^9^ CFU/mL.

For the oral phase, 2.5 mL of the yeast suspension was mixed with 2.0 mL SSF. Salivary amylase was omitted because the systems were starch-free. The pH was adjusted to 7.0 ± 0.2, the final volume was set to 5 mL with Milli-Q water, and the samples were incubated for 2 min at 37 °C with agitation (100 rpm). For the gastric phase, the oral bolus was mixed with 4 mL SGF, gastric lipase (25 U/mL), and pepsin (2000 U/mL), adjusted to 3 ± 0.2, the final volume was set to 10 mL, and the mixture was incubated for 2 h at 37 °C with agitation. For the intestinal phase, the gastric chyme was mixed with SIF, pancreatin (200 U/mL), and bile solution as described in the modified method. The pH was adjusted to 7.0 ± 0.2, the final volume was set to 20 mL, and the samples were incubated for 2 h at 37 °C with agitation.

Calcium chloride (0.3 M) was added to each phase according to the protocol specifications. At the end of each stage, aliquots (100 µL) were collected, serially diluted, and plated on YPD agar for viable cell enumeration. Plates were incubated at 30 °C for 48 h. Colony counts were used to determine viable populations (CFU/mL), from which survival percentages were calculated.

### 2.6. Tolerance to Bile Salt of Cocoa-Derived Yeast Strains

The growth response of yeast strains under bile salts was evaluated using the modified method of Pedersen et al. [34]. Yeast strains were cultivated in YPD broth for 24 h at 30 °C, harvested by centrifugation at 5000 rpm for 15 min at 4 °C, and the resulting pellets were resuspended in YPD broth supplemented with 0.1%, 0.3%, and 0.5% Oxgall (Merck, Rahway, NJ, USA). Cell suspensions were incubated at 37 °C for 24 h. Optical density at 600 nm was measured after 1, 2, 4, and 24 h of incubation, with uninoculated medium as the blank. OD_600_ values were used as a comparative indicator of biomass development under bile salt stress during the assessment.

### 2.7. Auto-Aggregation Capacity of Cocoa-Derived Yeast Strains

The auto-aggregation capacity of the evaluated yeast strains was determined using a modified method based on Simões et al. [35]. Yeast cultures were grown in YPD broth at 30 °C for 24 h, harvested by centrifugation (5000 rpm, 15 min, 4 °C), and the resulting pellets were washed twice with 3 mL of phosphate-buffered saline (PBS, 0.5 M, pH 7). Cell suspensions were then adjusted to an optical density of approximately OD_600_ = 0.9 using PBS as the blank. Suspensions were incubated at 37 °C without agitation. Optical density at 600 nm (OD_600_) was measured at 2 h and 4 h of incubation. The auto-aggregation percentage was calculated using Equation (1)

(1) $$Auto-aggregation\;\left(\%\right)=\left[1-(\frac{A_{t}}{A_{0}})\right]\times 100$$

where *A*_t_ represents the OD_600_ nm after incubation and *A*_0_ represents the OD_600_ nm at 0 h.

### 2.8. Coaggregation Assay of Cocoa-Derived Yeast Strains

To quantify the coaggregation properties (yeast–bacterial cell interactions), the methodology of Pessoa et al. [36] was used. Yeast cells were cultured in YPD broth at 30 °C for 24 h, harvested by centrifugation (5000 rpm, 15 min, 4 °C), washed twice with phosphate-buffered saline (PBS, 0.5 M, pH 7), and resuspended in the same buffer. Cell suspensions were adjusted to an OD_600_ of 0.9. Indicator bacterial strains, including *Escherichia coli* CDBB-B-964, *Listeria innocua* CDBB-B-1866, *Bacillus cereus* CDBB-B-1425, and *Micrococcus luteus* CDBB-B-72, were cultured under appropriate conditions and prepared similarly. These bacteria were selected as non-pathogenic indicator strains and are commonly used as model organisms due to their phylogenetic or functional relationship with clinically relevant or spoilage-associated bacteria.

For the coaggregation assay, equal volumes (2 mL) of yeast and bacterial suspensions were mixed and vortexed for 10 s. Control tubes containing only yeast or bacterial suspensions were prepared. Mixtures were incubated at 30 °C for 4 h without agitation. After incubation, absorbance was measured at 600 nm. Coaggregation percentage was calculated using Equation (2), whereas a decrease in absorbance reflects cell–cell interaction and aggregation between yeast and bacterial cells.

(2) $$Coaggregation\;\left(\%\right)=\left[\frac{\frac{Ax+Ay}{2}-A\left(x+y\right)}{\frac{Ax+Ay}{2}}\right]\times 100$$

where *Ax* and *Ay* indicate the absorbance of strains in the control tubes, and *A*(*x* + *y*) indicates the absorbance of the mixture’s tubes.

### 2.9. Cell Surface Hydrophobicity (MATH) Capacity of Cocoa-Derived Yeast Strains

Cell surface hydrophobicity was evaluated using the Microbial Adhesion to Hydrocarbons (MATH) method, as described by Gut et al. [37], with minor modifications. Yeast cultures incubated in YPD broth at 30 °C for 24 h were harvested by centrifugation (5000 rpm, 15 min, 4 °C), washed twice with phosphate-buffered saline (PBS, 0.5 M, pH 7), and resuspended in the same buffer. Cell suspensions were adjusted to an optical density at 600 nm (OD_600_) of approximately 0.9 using PBS as the blank. Aliquots of 3 mL of the standardized yeast suspension were transferred to test tubes, and 0.6 mL of chloroform was added. The mixtures were vortexed for 10 min and then incubated at 37 °C for 1 h to allow phase separation. After incubation, the optical density of the aqueous phase was measured at 600 nm. Hydrophobicity percentage was calculated from the reduction in OD_600_, where a decrease in absorbance indicates cell adhesion to the hydrocarbon phase (chloroform). The percentage of hydrophobicity was calculated using Equation (3)

(3) $$Hydrophobicity\;\left(\%\right)=\left[\frac{A_{0}-A}{A_{0}}\right]\times 100$$

where *A*_0_ and *A* denote the absorbance before and after chloroform extraction, respectively.

### 2.10. Bile Salt Hydrolase Activity of Cocoa-Derived Yeast Strains

Bile salt hydrolase (BSH) activity was qualitatively evaluated using the method described by Hernández-Gómez [38]. Yeast strains were inoculated onto sterile 6 mm diameter filter disks, which were placed on YPD agar supplemented with 0.5 g/L bile salts and 0.37 g/L CaCl_2_. Plates were incubated at 37 °C for 72 h. After incubation, the presence of opaque precipitation halos around the disks was recorded as a positive indication of BSH activity, whereas the absence of halos was considered a negative result. This assay was used as an initial qualitative screening and does not provide a quantitative measure of enzymatic activity.

### 2.11. Qualitative Response of Cocoa-Derived Yeast Strains to Antifungal Agents

The response of cocoa-derived yeast strains to selected antifungal compounds was evaluated using a modified method based on Fernández-Pacheco [39]. This assay was conducted as a preliminary qualitative screening of growth inhibition and was not intended as a standardized clinical susceptibility test. Yeast suspensions were uniformly spread onto agar plates using the microbial lawn technique. Subsequently, 5-μL aliquots of each antifungal formulation were applied to the inoculated agar surface. Plates were incubated at 37 °C for 48 h. After incubation, inhibition zones surrounding each antifungal deposit were examined as an indicator of growth suppression. Growth response was assessed by measuring the diameter (mm) of zones of total inhibition (absence of visible growth) and partial inhibition (reduced growth). The antifungal agents tested were commercial formulations of Fluconazole (150 mg mL^−1^) (Medimart^®^, Walmart, Bentonville, AR, USA), Ciclopirox (1 mg mL^−1^) (Bonnetril^®^, Glenmark, Elmwood Park, NJ, USA), Nystatin (21 mg mL^−1^) (Medimart^®^, Walmart), and Clotrimazole (10 mg mL^−1^) (Canesten^®^, Bayer, Leverkusen, Germany).

### 2.12. Statistical Analysis

All experiments were independently repeated and performed in triplicate. Results were expressed as the mean ± standard deviation (SD). Statistical analyses were performed using NCSS 2021 Statistical (Kaysville, UT, USA, http://ncss.com/software/ncss, accessed on 24 September 2025). Before analysis of variance, data were assessed for normality and homogeneity of variance. Differences among treatments were evaluated using one-way or two-way analysis of variance (ANOVA), depending on the experimental design. When significant differences were detected, means were compared using the Tukey–Kramer multiple comparisons test. A significance level of *p* ≤ 0.05 was used throughout this study.

## 3. Results and Discussion

### 3.1. Hemolytic Activity of Cocoa-Derived Yeast Strains

Safety is a fundamental criterion for selecting microorganisms with probiotic potential, as strains that exhibit virulence factors are not suitable for further application [39]. Among the in vitro assays commonly used for preliminary safety assessment, hemolytic activity remains a widely accepted indicator [40]. In this study, none of the yeast strains isolated from cocoa fermentation exhibited hemolytic activity under the evaluated conditions, indicating the absence of this virulence-related trait. These results are consistent with previous studies describing non-hemolytic behavior in most food-associated yeast strains, although hemolytic activity has been occasionally reported in certain species [41,42]. Therefore, all evaluated *S. cerevisiae* strains were suitable for further analysis.

### 3.2. Tolerance to Temperature of Cocoa-Derived Yeast Strains

**Figure 1:**
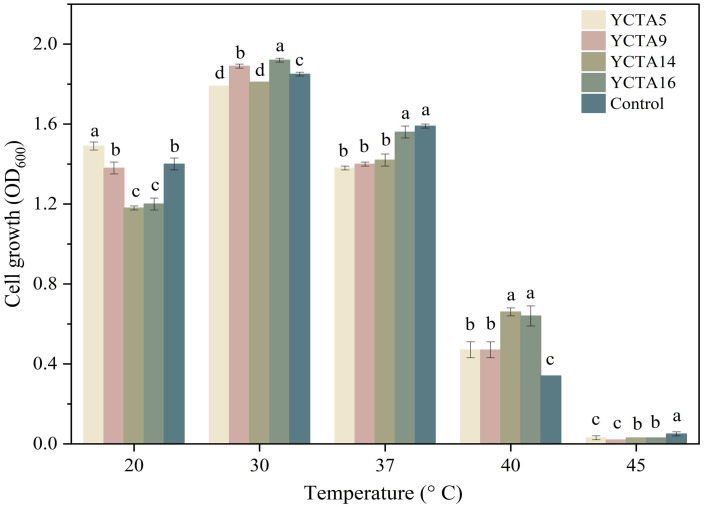
Temperature tolerance of cocoa-derived yeast strains. Growth (OD_600_) of *Saccharomyces cerevisiae* strains and *Saccharomyces boulardii* after 24 h at different temperatures. Values are mean ± SD (n = 3). Different letters indicate significant differences (*p* < 0.05, Tukey–Kramer test).

Temperature is a key factor in evaluating microorganisms with traits associated with probiotic functionality, particularly their ability to grow at physiological temperature (37 °C) and remain metabolically active [43,44]. All evaluated yeast strains exhibited growth (OD_600_), used here as a comparative indicator of tolerance, at 20, 30, and 37 °C (Figure 1). The highest growth was observed at 30 °C, with *S. cerevisiae* YCTA16 showing significantly higher values, followed by YCTA9 and the commercial probiotic strain (*S. boulardii*). At 37 °C, growth decreased across all yeast strains; however, *S. boulardii* and *S. cerevisiae* YCTA16 maintained significantly higher OD_600_ values that did not differ significantly from each other. At 40 °C, a marked reduction in growth was observed for all strains, although *S. cerevisiae* YCTA14 and YCTA16 retained relatively higher values. No measurable growth was detected at 45 °C (OD_600_ < 0.1). These results indicate a progressive decline in growth as temperature increases beyond optimal conditions.

Similar trends have been reported for yeast strains isolated from fermented foods, which generally grow at 37 °C but show reduced tolerance at higher temperatures [20,41]. The ability of cocoa-derived yeast strains to maintain growth at 37 °C suggests adaptation to physiologically relevant conditions, whereas the lack of growth at 45 °C indicates thermal limits beyond those of typical host-associated environments. Overall, these results support including the evaluated yeast strains in an initial assessment and highlight differences in thermal tolerance among strains.

### 3.3. Tolerance to pH of Cocoa-Derived Yeast Strains

**Figure 2:**
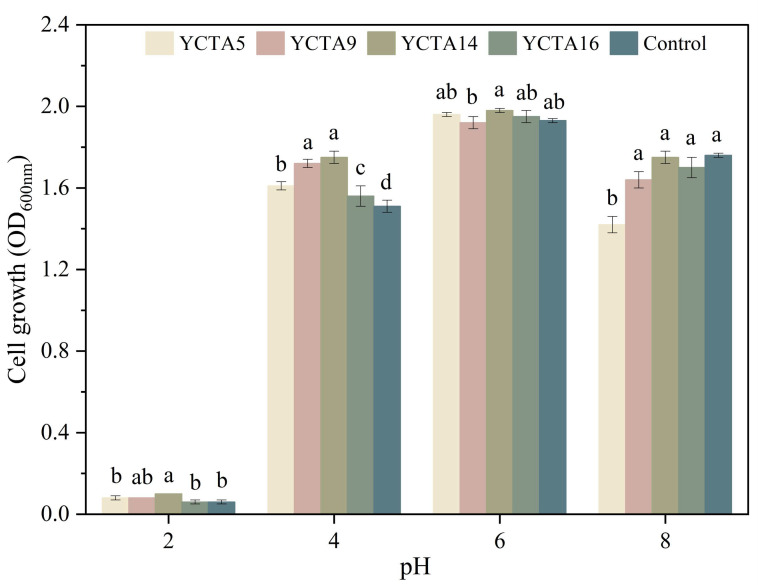
pH tolerance of cocoa-derived yeast strains. Growth (OD_600_) of *Saccharomyces cerevisiae* strains and *Saccharomyces boulardii* after 24 h of incubation at 30 °C at different pH values. Values are mean ± SD (n = 3). Different letters indicate significant differences (*p* < 0.05, Tukey–Kramer test).

All evaluated yeast strains exhibited OD_600_ values at pH 4, 6, and 8 (Figure 2). The highest growth occurred at pH 6, with *S. cerevisiae* YCTA9 showing slightly lower growth than the other yeasts evaluated. At pH 2, none of the strains, including the commercial probiotic, exhibited measurable growth (OD_600_ < 0.1). Tolerance to acidic conditions is considered a relevant criterion in the exploratory characterization of microorganisms with traits associated with probiotic functionality, as strains are expected to withstand low pH conditions encountered in the gastrointestinal tract [43,45]. However, OD_600_ measurements provide an indirect estimate of biomass and do not directly reflect cell viability under these conditions. Although OD_600_ measurements are useful for comparative screening purposes, they may not fully reflect viable cell populations under stress conditions.

Previous studies have reported that some food-associated yeasts, including those isolated from cocoa fermentation, can tolerate low pH values, although growth at pH 2 is often limited and strain-dependent [23,32]. In the present study, the absence of measurable growth at pH 2 suggests reduced tolerance to prolonged exposure to highly acidic conditions. This reduced tolerance may be associated with acid-induced cellular stress mechanisms, including membrane damage, intracellular acidification, and limited proton extrusion capacity, which have been described in yeast under low-pH conditions [45,46,47].

These results suggest a potential limitation in sustaining metabolic activity under extreme gastric conditions and underscore the need for an in-depth evaluation using complementary viability assays. Nevertheless, this observation should be interpreted in the context of the initial assessment, in which the lack of growth at pH 2 does not preclude further study but rather identifies a constraint for future functional assessment.

### 3.4. In Vitro Survival Under Simulated Gastrointestinal Conditions of Cocoa-Derived Yeast Strains

Survival under simulated gastrointestinal conditions was evaluated using a three-stage in vitro digestion model based on the INFOGEST 2.0 protocol (Section 2.5). Under simulated gastrointestinal tract (GIT) conditions, all yeast strains exhibited high initial concentrations (9 log CFU/mL) that progressively declined during digestion. A slight decrease was noted during the oral phase, while the most significant reduction (2–3 log units) happened during the gastric phase, highlighting this stage as the main stress point. Among the strains tested, *S. cerevisiae* YCTA14 exhibited a relatively lower loss of viability.

**Figure 3:**
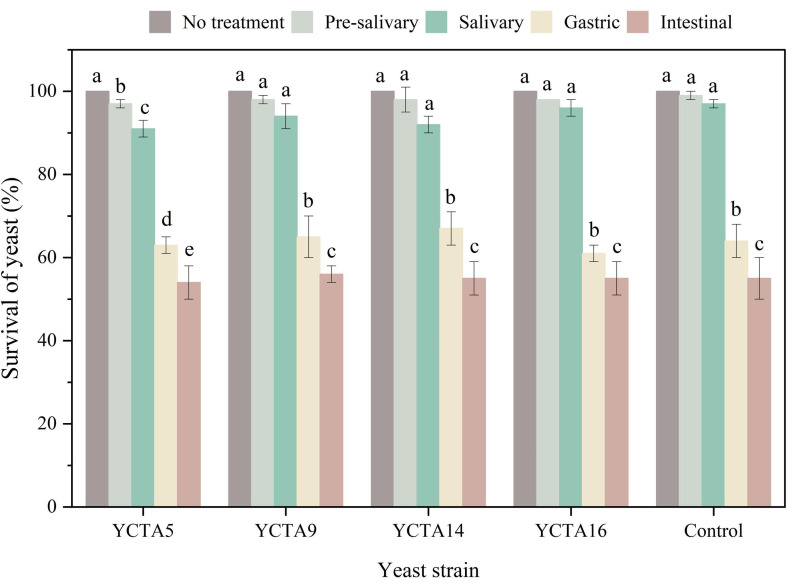
Survival of yeast strains under simulated gastrointestinal (GIT) conditions. Survival (%) of *S. cerevisiae* strains and *S. boulardii* after sequential exposure to simulated gastrointestinal phases. Values are mean ± SD (n = 3). Different letters indicate significant differences (*p* < 0.05; Tukey–Kramer test).

During the intestinal phase, viability further decreased to approximately 5 log CFU/mL, with no significant differences among strains, including *S. boulardii*. Survival percentages confirmed that the gastric phase had the greatest impact on cell viability (Figure 3), followed by a moderate additional reduction during the intestinal stage. The pronounced loss of viability during the gastric phase is consistent with the lack of growth observed at pH 2, indicating that acid stress likely plays a key role in limiting survival under simulated gastrointestinal conditions. However, this relationship should be interpreted with caution, as OD_600_ measurements reflect biomass development rather than direct cell viability.

These results highlight a potential limitation in maintaining viability under simulated gastric stress. Although final concentrations were below the commonly suggested threshold for probiotic functionality (10^6^ CFU/g or mL) [48], these findings should be interpreted as the exploratory assessment results. The observed reduction in viability indicates a constraint that could be addressed through strategies such as microencapsulation or formulation approaches to improve gastrointestinal survival [49]. Additionally, in vitro digestion models represent simplified systems and may not fully replicate in vivo conditions, including dynamic physiological factors [50]. Overall, these results are consistent with previous studies reporting reductions in yeast strain viability under simulated gastrointestinal conditions, supporting the need for further optimization and validation of functional traits [51].

### 3.5. Tolerance to Bile Salt of Cocoa-Derived Yeast Strains

All evaluated yeast strains exhibited cell growth (OD_600_) in YPD medium supplemented with 0.1% bile salt after 24 h of incubation (Table 1). Cocoa-derived strains showed higher OD_600_ values than the reference strain (*S. boulardii*), with no significant differences among *S. cerevisiae* YCTA5, YCTA9, and YCTA16. At higher bile salt concentrations (0.3 and 0.5%), no measurable growth was observed for any of the evaluated yeast strains (OD_600_ ≤ 0.1), indicating a strong inhibitory effect at these levels.

Bile salt tolerance is considered a relevant selection criterion in the initial characterization of microorganisms with probiotic potential. These compounds are present in the intestinal environment at concentrations that typically range from 0.3% to 0.6% [52,53]. Despite this, tolerance levels are strain-dependent and vary widely among yeast species.

The lack of growth at concentrations of ≥0.3% suggests a limited capacity to withstand bile salt stress under the tested conditions. These findings are consistent with previous studies reporting that some food-associated yeast strains maintain growth at low bile concentrations, but exhibit reduced tolerance at higher bile concentrations [34,54]. Furthermore, Wulan et al. [41] reported that cocoa-derived yeast showed growth at higher bile concentrations, highlighting the variability among strains. Bile salts can disrupt membrane integrity and cellular homeostasis in yeasts. Therefore, the limited growth observed above 0.1% may reflect strain-dependent differences in membrane lipid composition and stress response systems [55,56]. These results indicate a potential limitation in maintaining metabolic activity under physiologically relevant bile conditions. This observation should be treated as a constraint for further optimization rather than as grounds for excluding this cocoa-derived yeast strain from subsequent evaluation.

### 3.6. Auto-Aggregation Properties of Cocoa-Derived Yeast Strain

**Figure 4:**
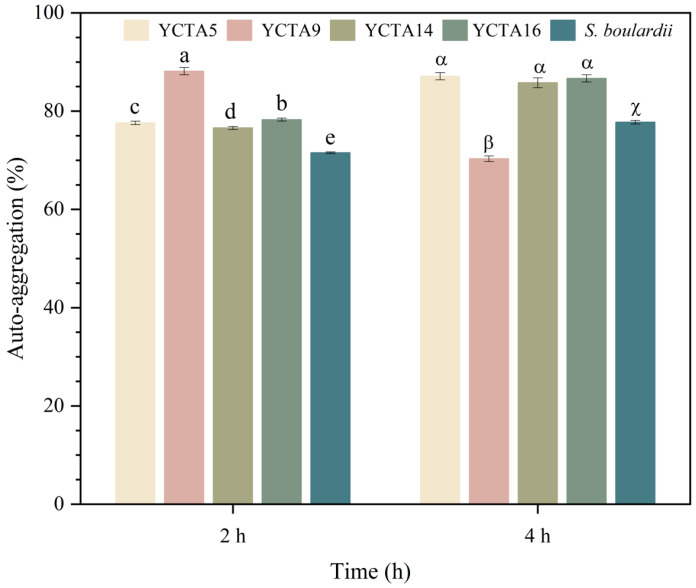
Auto-aggregation capacity of cocoa-derived yeast strains. Auto-aggregation (%) of *Saccharomyces cerevisiae* strains and *Saccharomyces boulardii*. Values represent mean ± SD (n = 3). Different lowercase letters indicate significant differences among strains at 2 h, whereas different Greek letters indicate significant differences at 4 h (*p* < 0.05, Tukey–Kramer test).

All cocoa-derived yeast strains exhibited high auto-aggregation values (>70%) at both incubation times (2 and 4 h), as shown in Figure 4. At 2 h, *S. cerevisiae* YCTA9 showed the highest percentage, followed by YCTA16, whereas *S. boulardii* showed the significantly lowest values. After 4 h of incubation, the *S. cerevisiae* YCTA5, YCTA14, and YCTA16 showed increased auto-aggregation values, while the *S. cerevisiae* YCTA9 exhibited a slight decrease compared to its performance at 2 h. Overall, all cocoa-derived strains maintained higher auto-aggregation levels than the commercial probiotic strain.

Auto-aggregation is a surface-related property associated with microbial interactions and potential adhesion to host tissues, and it is commonly considered a relevant trait in the first-stage evaluation of microorganisms with probiotic functionality. Higher auto-aggregation percentages may reflect an increased capacity for cell–cell interactions; however, this property alone is insufficient to infer probiotic functionality. In addition, yeasts differ structurally from probiotic bacteria in cell size and surface composition, which may influence their aggregation behavior in vitro. These traits may be associated with the presence of mannoproteins and cell wall polysaccharides, which play key roles in cell–cell interactions and surface-related properties [57,58,59,60]. Based on commonly used criteria, auto-aggregation values < 30% are considered low, 30–60% intermediate, and >60% high [23,61]. Accordingly, all evaluated yeast strains in this study can be classified as exhibiting high auto-aggregation. These results are consistent with previous reports on yeast strains from fermented foods, particularly cocoa fermentation [37,41,62], and support their inclusion in an initial screening.

### 3.7. Coaggregation Capacity of Cocoa-Derived Yeast Strain

**Figure 5:**
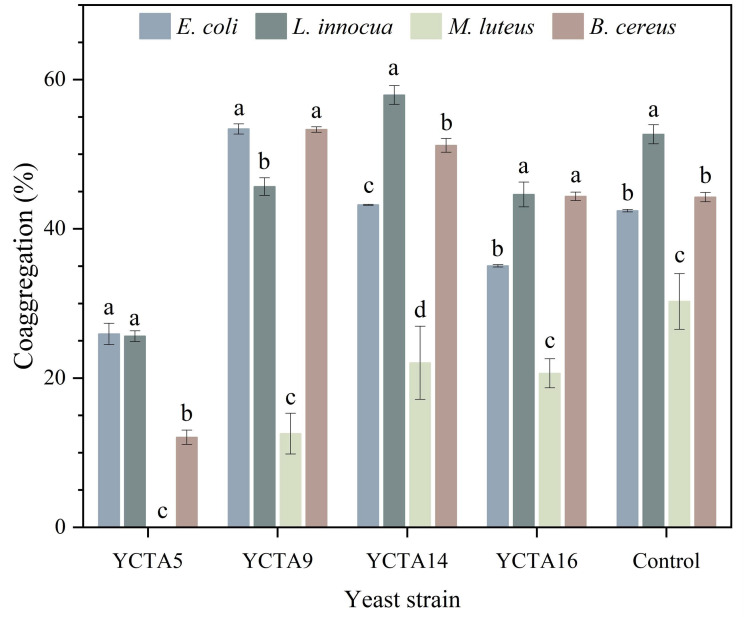
Coaggregation capacity of cocoa-derived yeast strains with bacterial indicators. Coaggregation (%) of *Saccharomyces cerevisiae* strains and *Saccharomyces boulardii* (control) with non-pathogenic indicator bacterial strains after 4 h of incubation. Values represent mean ± SD (n = 3). Different letters indicate significant differences (*p* < 0.05, Tukey–Kramer test).

Coaggregation refers to the ability of microorganisms to interact with other microbial cells and is associated with processes of microbial interaction that may contribute to competitive exclusion in complex ecosystems [59,63,64]. In the present study, the coaggregation capacity of yeasts against non-pathogenic indicator bacteria (*E. coli* CDBB-B-964, *L. innocua* CDBB-B-1866, *M. luteus* CDBB-B-72, and B. cereus CDBB-B-1425) was evaluated (Figure 5).

The results showed variable coaggregation capacities depending on both the yeast strain and the bacterial species. Overall, *S. cerevisiae* YCTA9 and YCTA14 exhibited higher coaggregation values against *E. coli* and *B. cereus*, with values comparable to the reference probiotic strain *S. boulardii*. In contrast, lower coaggregation values were observed with *M. luteus*, indicating reduced interaction with the bacterium.

These findings indicate that coaggregation capacity is both strain- and bacterium-dependent. In addition, structural differences between yeast and non-pathogenic indicator bacteria, particularly in cell size and surface composition, may influence coaggregation behavior. This interaction may be associated with cell wall components such as mannoproteins and polysaccharides, which are involved in cell–cell recognition and adhesion [59,65,66].

Similar variability has been reported in yeast strains from fermented foods, including those involved in cocoa fermentation, with coaggregation values varying widely across species and experimental conditions [23,35,56,67]. Although some strains exhibited coaggregation values comparable to those of the commercial probiotic yeast, these results should be interpreted as indicative of potential microbial interactions rather than as direct evidence of probiotic functionality. Nevertheless, this property alone is insufficient to infer the ability to prevent pathogen colonization. Therefore, the evaluated strains should be considered candidates for further investigation within a preliminary framework, and additional studies are required to confirm the functional relevance of these interactions under physiologically relevant conditions.

### 3.8. Cell Surface Hydrophobicity (MATH) Capacity of Cocoa-Derived Yeast Strain

Cell surface hydrophobicity is a surface-related property associated with microbial interactions and has been proposed as an indirect indicator of adhesion potential. Adhesion of microorganisms with traits associated with probiotic functionality involves physicochemical interactions, such as van der Waals forces, hydrophobic interactions, and hydrogen bonding [68,69,70], and depends on cell-surface composition and environmental conditions. Surface properties such as hydrophobicity and auto-aggregation are therefore evaluated as indicators of microbial interaction potential under in vitro conditions. Nevertheless, these parameters do not fully represent the complexity of adhesion processes in vivo, which are influenced by additional factors such as mucus composition, host receptors, and gastrointestinal dynamics. Consequently, they are considered part of an exploratory characterization approach rather than definitive evidence of adhesion capacity. These traits may be linked to mannoproteins and cell wall polysaccharides involved in cell–cell interactions [59].

**Figure 6:**
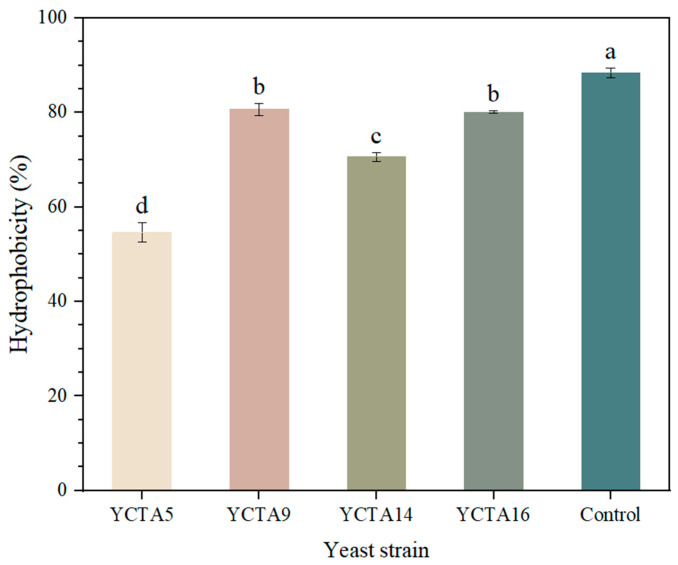
Cell surface hydrophobicity of cocoa-derived yeast strains determined by MATH assay. Hydrophobicity (%) of *Saccharomyces cerevisiae* strains and *Saccharomyces boulardii* evaluated by MATH assay using chloroform as the solvent phase. Values represent mean ± SD (n = 3). Different letters indicate significant differences (*p* < 0.05, Tukey–Kramer test).

In this study, the commercial probiotic strain *S. boulardii* exhibited the highest value (88.4%), followed by *S. cerevisiae* YCTA9 and YCTA16 (~80%), with no significant differences among them (Figure 6). In contrast, *S cerevisiae* YCTA5 showed the lowest value (55%). According to commonly used criteria, hydrophobicity values < 20% are considered low, 20–50% moderate, and >50% high [71]. Based on this classification, all evaluated yeast strains can be considered highly hydrophobic. The values obtained are consistent with previous reports of yeast strains evaluated using the MATH assay, including cocoa-derived yeast strains, although variability among studies is common due to differences in solvent polarity and strain-specific surface properties [23,53,69].

Although hydrophobicity and aggregation properties were favorable, these surface-related traits alone are insufficient to support classification as probiotic potential when survival under acidic and bile conditions is limited. Probiotic functionality requires a combination of safety, gastrointestinal robustness, and demonstrated host benefit. Therefore, the evaluated strains should be considered preliminary candidates requiring further validation.

### 3.9. Bile Salt Hydrolase Activity of Cocoa-Derived Yeast Strains

Qualitative analysis indicated that none of the cocoa-derived yeast strains exhibited bile salt hydrolase (BSH) activity. The absence of BSH-activity observed in this study is consistent with the limited growth of these strains at higher bile salt concentrations, as shown in the bile tolerance assay. Previous studies, such as those by Fadda et al. [72], have reported BSH activity in *Kluyveromyces* lactis and *K. marxianus*, suggesting that this trait may vary by species and strain origin.

Bile salt deconjugation has been proposed as a mechanism involved in microbial adaptation to gastrointestinal environments and has also been associated with cholesterol metabolism in some studies [73]. Nonetheless, the presence or absence of BSH-activity alone is not sufficient to determine bile tolerance or probiotic functionality. Therefore, the lack of detectable BSH activity in the strains evaluated should be interpreted as a preliminary evaluation result. Although this may be a limitation, it does not preclude further investigation of these strains, as alternative mechanisms may contribute to bile tolerance and functional performance under gastrointestinal conditions.

### 3.10. Qualitative Response of Cocoa-Derived Yeast Strains to Antifungal Agents

The response of cocoa-derived yeast strains to select antifungal compounds was evaluated using a qualitative disk diffusion assay. Under the tested conditions, no inhibition zones were observed for any of the evaluated yeast strains, including the commercial probiotic strain *S. boulardii* (Table 2), indicating no detectable inhibition. It is important to note that this assay provides a preliminary qualitative assessment and does not allow definitive classification of antifungal resistance, which requires standardized minimum inhibitory concentration (MIC) determinations. Previous studies have reported variable responses among yeast strains across species, highlighting the strain-dependent nature of antifungal response [74,75,76]. The absence of inhibition under the tested conditions does not imply antifungal resistance, and further studies using standardized quantitative methods are required to accurately determine susceptibility profiles.

## 4. Conclusions

This study provides a preliminary characterization of autochthonous *Saccharomyces cerevisiae* strains isolated from Mexican cocoa bean fermentation. The evaluated strains exhibited favorable characteristics, including growth at 37 °C, absence of hemolytic activity, high auto-aggregation and hydrophobicity, and moderate coaggregation capacity with *E. coli*, *L. innocua*, and *B. cereus*, particularly in YCTA9, YCTA14, and YCTA16. These properties are considered relevant indicators of potential interaction with the host environment. However, key limitations were identified, including the absence of growth under highly acidic conditions (pH 2), limited tolerance to bile salts at physiologically relevant concentrations, and reduced survival during simulated gastrointestinal transit. These findings indicate that the evaluated strains do not fully meet key physiological criteria for probiotic functionality under the tested conditions. Therefore, the strains should be regarded as preliminary candidates for further evaluation rather than validated probiotics. Mexican cocoa bean fermentation represents an underexplored reservoir of yeast biodiversity with potential biotechnological potential. Future studies should focus on improving gastrointestinal tolerance and validating functional traits using in vivo models.

## Figures and Tables

**Table 1:** Bile salt tolerance of cocoa-derived yeast strains.

Yeast Strain	Incubation Time			
	1 h	2 h	4 h	24 h
*S. cerevisiae* YCTA5	0.11 ± 0.01 ^b^	0.12 ± 0.01 ^b^	0.12 ± 0.02 ^b^	1.31 ± 0.02 ^aA^
*S. cerevisiae* YCTA9	0.11 ± 0.01 ^b^	0.12 ± 0.01 ^b^	0.13 ± 0.04 ^b^	1.38 ± 0.00 ^aA^
*S. cerevisiae* YCTA14	0.11 ± 0.00 ^b^	0.13 ± 0.01 ^b^	0.13 ± 0.03 ^b^	1.15 ± 0.01 ^aB^
*S. cerevisiae* YCTA16	0.11 ± 0.01 ^b^	0.12 ± 0.01 ^b^	0.13 ± 0.01 ^b^	1.44 ± 0.07 ^aA^
Control (*S. boulardii*)	0.14 ± 0.01 ^b^	0.11 ± 0.01 ^b^	0.09 ± 0.01 ^b^	0.62 ± 0.09 ^aC^

Values are mean ± SD (n = 3). Different lowercase letters indicate significant differences across time points within each strain, while uppercase letters indicate differences among strains (*p* ≤ 0.05, Tukey–Kramer test).

**Table 2:** Qualitative response of cocoa-derived yeast strains to antifungal agents.

Yeast Strain	Antifungal Agents			
	Fluconazole (150 mg/mL)	Clotrimazole (10 mg/mL)	Nystatin (21 mg/mL)	Ciclopirox (1 mg/mL)
*S. cerevisiae* YCTA5	No inhibition zone detected	No inhibition zone detected	No inhibition zone detected	No inhibition zone detected
*S. cerevisiae* YCTA9	No inhibition zone detected	No inhibition zone detected	No inhibition zone detected	No inhibition zone detected
*S. cerevisiae* YCTA14	No inhibition zone detected	No inhibition zone detected	No inhibition zone detected	No inhibition zone detected
*S. cerevisiae* YCTA16	No inhibition zone detected	No inhibition zone detected	No inhibition zone detected	No inhibition zone detected
*S. boulardii* (Control)	No inhibition zone detected	No inhibition zone detected	No inhibition zone detected	No inhibition zone detected

Results correspond to a qualitative assay and should not be interpreted as standardized clinical resistance profiles.

## Data Availability

The original contributions presented in this study are included in the article. Further inquiries can be directed to the corresponding authors.
